# The Role of microRNA Markers in the Diagnosis, Treatment, and Outcome Prediction of Spinal Cord Injury

**DOI:** 10.3389/fsurg.2016.00056

**Published:** 2016-11-08

**Authors:** Nikolay L. Martirosyan, Alessandro Carotenuto, Arpan A. Patel, M. Yashar S. Kalani, Kaan Yagmurlu, G. Michael Lemole, Mark C. Preul, Nicholas Theodore

**Affiliations:** ^1^Department of Neurosurgery, Barrow Neurological Institute, St. Joseph’s Hospital and Medical Center, Phoenix, AZ, USA; ^2^Division of Neurosurgery, University of Arizona, Tucson, AZ, USA; ^3^College of Medicine – Phoenix, University of Arizona, Phoenix, AZ, USA

**Keywords:** biomarker, gene expression, microRNA, miRNA, regeneration, spinal cord injury

## Abstract

Spinal cord injury (SCI) is a devastating condition that affects many people worldwide. Treatment focuses on controlling secondary injury cascade and improving regeneration. It has recently been suggested that both the secondary injury cascade and the regenerative process are heavily regulated by microRNAs (miRNAs). The measurement of specific biomarkers could improve our understanding of the disease processes, and thereby provide clinicians with the opportunity to guide treatment and predict clinical outcomes after SCI. A variety of miRNAs exhibit important roles in processes of inflammation, cell death, and regeneration. These miRNAs can be used as diagnostic tools for predicting outcome after SCI. In addition, miRNAs can be used in the treatment of SCI and its symptoms. Significant laboratory and clinical evidence exist to show that miRNAs could be used as robust diagnostic and therapeutic tools for the treatment of patients with SCI. Further clinical studies are warranted to clarify the importance of each subtype of miRNA in SCI management.

## Introduction

Acute spinal cord injury (SCI) is a devastating condition that affects mostly young and active individuals. It is estimated that approximately 180,000 persons around the world will experience some form of traumatic SCI every year (1–3). These injuries are particularly devastating because they can result in physically, socially, and financially burdensome consequences, such as quadriplegia and paraplegia. Over the past decade, we have gained a much better understanding of the biological mechanisms underlying the damage caused by acute SCI. This damage can be divided into two phases: primary and secondary (4, 5).

The primary phase of damage after SCI is characterized by the immediate loss of sensory, motor, and autonomic functions after a sheer, lacerating, or impact injury to the spinal cord or sudden compression of the spinal cord. The initial mechanical damage to the spinal cord primarily disrupts gray matter and microvasculature. In contrast, white matter, although damaged in the primary phase, is primarily damaged in the secondary phase (4). The secondary phase of damage begins seconds after SCI and continues for months after injury. Within seconds to minutes of injury, vascular and metabolic disturbances occur in the spinal cord. In a matter of minutes to hours, biochemical changes lead to altered lipid peroxidation and neurotransmitter accumulation. Within hours to weeks, cascades of inflammatory cells and evidence of apoptosis occur, and, in weeks to months, fiber tract disturbances occur due to demyelination and glial scar formation (4–6). The secondary phase of damage is the primary target for investigation and clinical therapeutics.

Despite advances in our understanding of the pathways activated and responsible for secondary injury after SCI, therapeutic advances for patients with SCI have lagged behind. So far, the “one-size-fits-all” approach to treating SCI has repeatedly come up short. Gaps in our knowledge are partially responsible for the lack of improvement in therapy. In addition, the inability to identify a *bona fide* biomarker that predicts which patients are likely to have a good or poor outcome after SCI has hindered delivery and implementation of new therapies. Because no two SCIs are alike, treatment approaches should be tailored to the nature, quality, and duration of the injury. Novel approaches to SCI treatment will thus involve a shift toward personalized medicine.

In recent years, investigators have begun to document the potential effects that microRNA (miRNA) sequences may have on the regulation of the processes involved in SCI (7–9). The miRNA sequences are small, unique, non-coding RNA fragments that form hairpin structures averaging 22 nucleotides in length. They are produced in the nucleus by RNA polymerase II and processed by a variety of proteins before entering the cytoplasm as pre-miRNA. In the cytoplasm, the enzyme coded for by the gene, dicer 1 ribonuclease type III, or *DICER1*, processes the pre-miRNA duplex into a single-stranded miRNA sequence. This single-stranded miRNA sequence is incorporated into an RNA silencing complex that then binds to an mRNA sequence to carry out a negative regulatory effect by either degrading or blocking translation in the targeted mRNA (8, 10, 11). This regulatory system has a powerful effect on mRNA levels, and, as a result, it influences protein expression levels (12). Because miRNAs do not need a perfectly complementary sequence to act upon an mRNA, investigations have found that a single miRNA sequence is able to target up to 200 mRNA transcripts (11).

The miRNAs have a powerful and important influence on the protein profile of a given cell. Since the discovery of the first miRNA sequence in 1993, thousands of unique miRNA sequences that play key regulatory roles in the human body have been identified (8). In particular, miRNAs have been identified as playing an important role in regulating neurogenesis and cortical development (13). The management of SCI will be dramatically improved if novel pathophysiological mechanisms of SCI and sensitive biomarkers to monitor them can be determined. Alterations in extracellular RNAs could be used to identify regulators of secondary injury cascades, and overall changes in RNA concentrations could be used to stratify patients into risk categories for secondary injury and could be predictive of patient outcomes – a form of personalized medicine for patients suffering from the effects of SCI.

In this article, we review the regulatory role of miRNAs in the cascade of events, after an acute SCI. We also discuss their temporal and spatial expression, as well as their potential role as therapeutic agents in the personalized treatment of patients with SCI.

## SCI Pathophysiology and miRNAs

### Astrogliosis

Normally, astrocytes regulate central nervous system (CNS) homeostasis by maintaining the blood–brain barrier (BBB) and the blood–spine barrier, directing neuronal migration, differentiation, and development, and providing materials for axonal growth or regeneration (14). When a patient suffers a significant injury to the spinal cord and suffers the effects of secondary trauma, the area near the injury undergoes astrogliosis. In the acute stages of astrogliosis (at approximately the third day after injury), hypertrophic astrocytes expressing glial fibrillary acidic protein (GFAP) and vimentin (*VIM*) surround the lesion site, where they keep out inflammatory leukocytes, release antioxidants, and initiate repair of the blood–spinal cord barrier. These effects are both protective and beneficial. In later stages of astrogliosis (at approximately 4–6 weeks after injury), astrocytes change from hypertrophic to hyperplastic, forming a glial scar by expressing chondroitin sulfate proteoglycans. This is a detrimental process that compresses the entire lesion site and prevents the beneficial self-rehabilitating and protective actions that were present in the acute hypertrophic phase (Table 1) (7, 14–19).

**Table 1:** **Functions of miRNAs in spinal cord injury pathophysiology^a^**.

miRNA	4 h	12 h	1 day	3 days	4 days	7 days	14 days	≥30 days	Regulation	Target genes	Function
miR-9 (20)		↓	↓	↓		↑			↑ miR-9	↓ *MCPIP1*	↓ Inflammation, ↓ apoptosis
miR-17-5 (21)									↑ miR-17-5p	↓ *CDKN1A*, ↓ RB1	↑ Astrocyte proliferation
miR-20 (7, 22)	↑	↑	↑	↑	↑				↑ miR-20a	↓ *NEUROG1*	↑ Oxidative stress, ↑ functional recovery
miR-21 (8, 9, 15, 16, 18, 23–31)	↑		↑	↑	↑	↑	↓	↑	↑ miR-21	↓ *GFAP*	↓ Astrogliosis
									↑ miR-21	↓ *FASLG*, ↓ *PTEN*	↓ Apoptosis
									↑ miR-21	↑ *MTOR*	↑ Axon sprouting
									↑ miR-21	↓ CACNA1C, ↓ CACNA2D1, ↓ CACNAB1	↓ Calcium signaling, ↓ neurotransmission
									↑ miR-21		↑ Neuropathic pain
									↑ miR-21		↑ Functional recovery
miR-29b (22)	↓	↓	↓	↓	↓				↓ miR-29b	↑ *BH3*	↑ Apoptosis
miR-103 (23, 24, 32–34)	↑					↑			↑ miR-103	↓ CACNA1C, ↓ CACNA2D1, ↓ CACNAB1	↓ Neuropathic pain
									↓ miR-103	↑ CACNA1C, ↑ CACNA2D1, ↑ CACNAB1	↑ Neuropathic pain
									↓ miR-103		↑ Inflammation
miR-124 (35–39)		↓	↓	↓	↓	↓			↑ miR-124	↓ *PTBP1*	↑ Neuronal expression, ↑ functional recovery, ↓ apoptosis
miR-125b (9, 40)									↑ miR-125b		↑ Axonal regeneration
miR-126 (41)			↓	↓		↓			↑ miR-126	↓ *VCAM1*	↓ Immune cell infiltration
										↓ *PIK3R2*	↑ Apoptosis
										↓ *SPRED1*	↑ Cell growth, ↓ angiogenesis
miR-145 (17, 24, 42)	↑	↑	↑	↑		↓		↓	↑ miR-145	↓ *GFAP*, ↓ *MYC*	↓ Astrogliosis
									↓ miR-145	↑ *SOD2*	↓ Oxidative stress
miR-146 (7, 18, 24, 43, 44)						↑	↑		↑ miR-146	↓ *IRAK1, TRAF6*	↓ Inflammation
									↑ miR-146		↓ Astrogliosis
miR-146a (7, 18, 43, 44)	↓		↑			↑			↑ miR-146a	↓ *IRAK1*	↓ Astrogliosis
									↑ miR-146a	↓ *CASP3*, ↓ *FAS/CD95*	↓ Apoptosis
									↑ miR-146a	↓ *IL6*, ↓ *COX2*	↓ Inflammation
									↓ miR-146a	↑ *IL6*, ↑ *COX2*	↑ Inflammation
									↓ miR-146a	↑ *CASP3*, ↑ *FAS/CD95*	↑ Apoptosis
miR-155 (45, 46)				↑	↑	↑	↑		↑ miR-155 ↓ miR-155	↓ *SOCS1* ↑ *GAP43*, ↑ *NEFH*	↑ IL-17 Expression, ↑ Th17 differentiation
miR-181 (47, 48)									↓ miR-181	↑ *TNF*	↑ Inflammation
									↓ miR-181		↑ Astrogliosis
miR-195 (49)									↑ miR-195	↑ *IL1B*; ↑ *TNF*	↑ Neuropathic pain
									↓ miR-195	↓ *IL1B*; ↓ *TNF*	↓ Neuropathic pain
miR-199a-3p (27)									↓ miR-199a-3p	↑ *MTOR*	↑ Axon regeneration
miR-200c (50)				↑		↑	↑		↑ miR-200c	↓ *FAP-1*	↑ Apoptosis
miR-210 (51)									↑ miR-210		↑ Axon growth, ↑ remyelination, ↑ angiogenesis, ↑ functional recovery
miR-218 (52)			↑	↑		↑	↑		↓ miR-218	↓ *JAK, STAT3, SOCS3*	↓ Pain behavior, ↓ inflammation
miR-219 (53)									↓ miR-219	↑ *ELOVL7*	↑ Demyelination
miR-223 (7, 54)			↑	↑		↑			↑ miR-223	↓ *GRIA2*, ↑ *BAX, CASP3*, ↓ *BCL2*	↑ Apoptosis, ↓ angiogenesis, ↓ functional recovery
									↑ miR-223		↑ Inflammation
miR-320 (55)									↓ miR-320	↑ *HSP20*	↑ Neuroprotection, ↑ functional recovery
miR-381 (56)									↑ miR-381	↑ *HES1*	↑ Neuronal differentiation, ↓ glial cell differentiation

*^a^An up arrow (↑) indicates upregulation; a down arrow (↓) indicates downregulation*.

**Figure 1:**
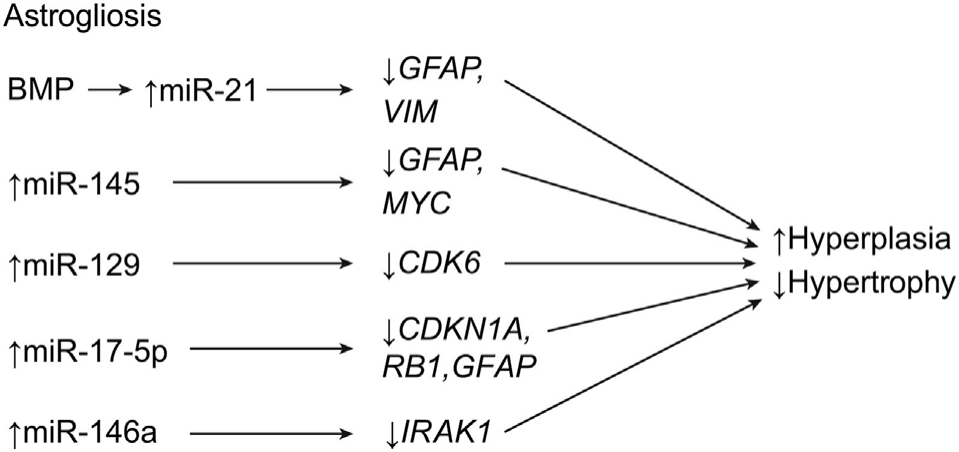
**Schematic for astrogliotic effects of miRNA expression after SCI**. The end result is a downregulation of astroglial swelling, and upregulation of cell division. *CDK6*, cyclin-dependent kinase 6; *CDKN1A* (*P21*), cycline-dependent kinase inhibitor 1A; *MYC*, V-myc avian myelocytomatosis viral oncogene homolog; *GFAP*, glial fibrillary acidic protein; *IRAK1*, interleukin-1 receptor-associated kinase-1; *MBP*, myelin basic protein; *RB1*, ginsenoside Rb1; *VIM*, vimentin. Used with permission from Barrow Neurological Institute, Phoenix, AZ, USA.

The process of astrogliosis has been shown to be associated with miRNAs, which play a pivotal role in the shift from hypertrophy to hyperplasia (7, 14–19). The miRNA miR-21 is highly expressed in astrocytes near and at the lesion site during astrogliosis, but not elsewhere (7). In addition, miR-21 is directly responsible for the shift from hypertrophy to hyperplasia, where it suppresses *GFAP* and *VIM* (Figure 1). The miR-21 is governed by bone morphogenic protein (BMP) signaling *via* the signal transducer and activator of the transcription 3 gene, *STAT3* (14–16, 19). Specifically, the BMP receptor type 1A and 1B genes, *BMPR1A* and *BMPR1B*, control astrogliosis by regulating miR-21 in opposing directions. *BMPR1A* downregulates miR-21 signaling, while *BMPR1B* upregulates it (7, 15, 16). These regulatory genes have become a target of interest for developing therapeutics. Knockout mice with suppressed miR-21 signaling maintain astrocyte hypertrophy, correlating with smaller lesion sites, less demyelination, greater axon regeneration, and an overall lower inflammatory response (7, 15, 16). Future treatment modalities could be geared toward preventing the shift to hyperplastic astrogliosis. The targets of these potential treatment modalities include the final processing of pre-miR-21 to its mature form, the formation of chondroitin sulfate proteoglycans, RNases that could suppress miR-21, and the suppression of *BMPR1B*.

Additional miRNAs involved in astrogliosis include miR-145, which is expressed in gray matter astrocytes during acute astrogliosis but which increases expression in astrocytes during the shift to hyperplasia. It has been shown to control the astrocytic cytoskeleton, where it affects cell growth and migration, in addition to negatively regulating *GFAP* and the cell growth gene, *MYC* (c-myc) (Table 1; Figure 1) (17). Strickland et al. have shown that approximately 30 miRNAs are altered by SCI (18). They report that miR-146a works with miR-21 to drive astrocyte hyperplasia, while miR 129-1 and miR 129-2 both inhibit the cyclin-dependent kinase gene, *CDK6*, and therefore prevent cell growth (Figure 1). The knockdown of these genes in SCI suggests a more conducive environment for hyperplastic astrogliosis (18).

In 2014, Hong et al. speculated that miR-17-5p is somehow involved in *p21 (CDKN1A)* regulation and promotes astroglial cell proliferation after injury by way of *DICER1* (Table 1) (21). Knocking out *DICER1* in mice caused miR-17-p5 to decrease *GFAP* expression while maintaining cell proliferation (Figure 1). This effect suggests that *DICER1* and miR-17-5p are directly involved in the maturation and proliferation of astrocytes. The knockdown of these components delayed astrocyte maturation and ultimately caused a failure to respond to the SCI cascade. These data further support the idea that selective manipulation of the astrogliotic response to SCI may be a key therapeutic strategy for SCI (21).

### Apoptosis

**Figure 2:**
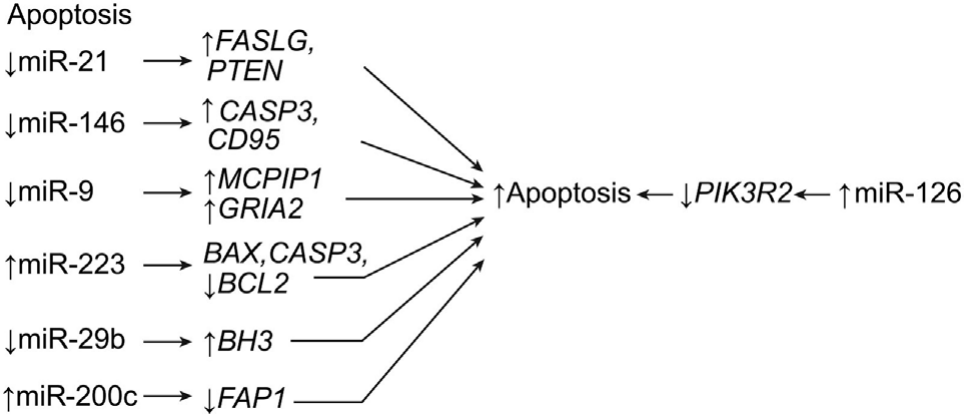
**Apoptotic effects of miRNA expression after SCI**. The result is apoptotic signaling. *BAX, BCL*2-associated X, apoptosis regulator; *BCL2*, B-cell chronic lymphocytic leukemia/lymphoma 2; *BH3, BCL2* homology domain 3; CASP3, caspase 3; *CD95*, also known as *FAS* receptor (cell surface death receptor), apoptosis antigen 1, and tumor necrosis factor receptor superfamily member 6; *FAP1*, Fas-associated phosphatase-1; *GRIA2*, glutamate ionotropic receptor AMPA (α-amino-3-hydroxy-5-methyl-4-isoxazolepropionic acid) type subunit 2; *MCPIP1*, monocyte chemotactic protein-induced protein 1; *PIK3R2*, phosphoinositide-3-kinase regulatory subunit 2; *PTEN*, phosphatase and tensin homolog. Used with permission from Barrow Neurological Institute, Phoenix, AZ, USA.

Apoptosis, or programed cell death, is a hallmark of SCI. Apoptosis can affect all cell types in the spinal cord, including glial cells. This is important when considering that SCI induces miRNA expression to either upregulate or downregulate apoptotic genes, depending on the target (Table 1) (7, 14, 23–25, 57–63). Among the miRNAs involved in this process, miR-21 has been shown to be one of the most dysregulated miRNAs after SCI (24, 25). As mentioned above, the shift from hypertrophy to hyperplasia in astrogliosis is heavily governed by miR-21, and the suppression of miR-21 is known to cause apoptosis. The miR-21 is a downregulator of the Fas ligand gene, *FASLG*, and the phosphatase and tensin homolog gene, *PTEN*, both of which promote apoptosis (Table 1; Figure 2) (7, 25, 58, 63). Strickland et al. demonstrated that miR-21 was significantly upregulated 4 days after SCI, only to be downregulated, relatively, by day 14 (18). This effect explains the transition from hypertrophy to hyperplasia that occurs 4–10 days after injury. This trend parallels that described by Liu et al., who reported that miR-21 levels were not significantly elevated at 10 days after SCI, but were still somewhat elevated (63). Although the suppression of miR-21 appears to have many beneficial effects related to astrogliosis, neuronal cell death has the opposite effect. Suppressing miR-21 using antagomir-21 increases neuronal deficits and lesion size in spinal cord tissue at 28 days after SCI (25). This effect is in contrast to findings of the previously mentioned study that found that the inhibition of miR-21 causes smaller lesion sizes and more effective recovery at 1–2 weeks after injury. However, both sets of data may be correct within their respective time frames.

Like miR-21, miR-146a is antiapoptotic as well as anti-inflammatory. However, it appears that, while miR-21 decreases in expression after 14 days, miR-146a remains constant in upregulation (Table 1) (18). This constant upregulation may indicate that miR-146a maintains the antiapoptotic state after the acutely reacting miR-21 has downregulated. In addition to miR-21 and miR-146a, miR-9 controls apoptosis by directly regulating the monocyte chemotactic protein-induced protein 1 gene, or *MCPIP1*, which is a known proapoptotic and macrophage-activating gene (Figure 2) (20). This postulated mi-9-controlled apoptosis is supported by the observation that, between days 1 and 7 after SCI, miR-9 was significantly downregulated in ventral horn motor neurons whereas *MCPIP1* was upregulated at the lesion site. However, by day 7, the miR-9 expression had increased, suppressing the expression of *MCPIP1*. Interestingly, *MCPIP1* also promotes GFAP, which is expressed by reactive astrocytes during astrogliosis (20). Thus, miR-9 appears to have a bimodal effect on SCI, such that its downregulation during acute stages allows for the expression of *GFAP* and the activation of astrocytes, while its upregulation at day 7 suggests a neuroprotective role of ventral motor horn cells. Considering that miR-21 plays a strong antiapoptotic role during acute SCI, miR-9 may work the opposite of miR-21, such that the downregulation of one is countered by the upregulation of the other. Further research is needed to observe the expression of miR-9 beyond 7 days in order to elucidate the relationship between these two miRNAs.

Other studies have shown that miR-223 is expressed in human and mouse hematopoietic systems (64). In SCI, miR-223 is temporally expressed, with increased peaks of expression at 1, 3, 7, and 14 days after SCI. Antagomir-223 treatment after SCI resulted in significantly lowered apoptotic cells, coinciding with downregulated *BAX* and *CASP3* and upregulated *BCL2*. This treatment preserved spinal cord tissue and significantly increased scores on the Basso, Beattie, and Bresnahan locomotor scale as early as 3 days after SCI (Table 1; Figure 2) (64).

In general, apoptosis after SCI is caused by either downregulation of miRNAs that target proapoptotic genes, such as caspase family genes, or upregulation of miRNAs that target antiapoptotic genes, such as *BCL2* or *MYC* (Table 1) (7, 23, 60–63). These regulatory effects provide multiple avenues for potential therapeutic strategies, which involve managing the balance of proapoptotic and antiapoptotic miRNAs. One such strategy was described by Liu et al. in 2010; in their study, a 5-day cycling exercise regimen within the first 10 days of injury significantly altered miRNA expression in mice (63). Their study showed that, with exercise, the antiapoptotic agents, miR-21 and *BCL2*, are upregulated, while proapoptotic agents miR-15b, *CASP7*, and *CASP9* are downregulated. These authors posit that exercise after SCI may stimulate beneficial antiapoptotic effects through the influence of miR-21 on the v-akt murine thymoma viral oncogene homolog 1 gene, *AKT1*, and on phosphatidylinositol triphosphate (Table 1) (63). Another potential therapy strategy is posttraumatic hypothermia and antisense silencing. Truettner et al. demonstrated that specific miRNAs are sensitive to posttraumatic hypothermia in brain lesions, which downregulates their apoptotic effects (59).

### Endogenous Antioxidant Systems and Neuroprotection

Another secondary effect of SCI, traumatic brain injury, or ischemia is the presence of reactive oxygen and nitrogen species (65). These molecules function to destroy cell and DNA structure, interfere with important cellular processes, and ultimately cause unintended cell death. The production of free radicals is caused by cytotoxic concentrations of glutamate (65, 66). During astrogliosis (the hypertrophic state), astrocytes build barriers against these reactive species, releasing antioxidants by way of the superoxide dismutase (SOD) family of genes and diverting glutamate excitotoxicity away from oligodendrocytes and neurons (14). However, during an oxidative crisis, miRNAs appear to exhibit functions that hinder the body’s protective response. For example, it has been observed that miR-21 strongly influences reactive oxygen species (ROS)-induced apoptosis during oxidative stress (67). When miR-21 was silenced, ROS-induced cell death was reduced in spinal cord neurons. The effect on spinal cord cells by miR-21 was analogous to that of free radicals (Table 1) (67). These data suggest that the antiapoptotic effects of miR-21 overexpression do not apply to neurons.

**Figure 3:**
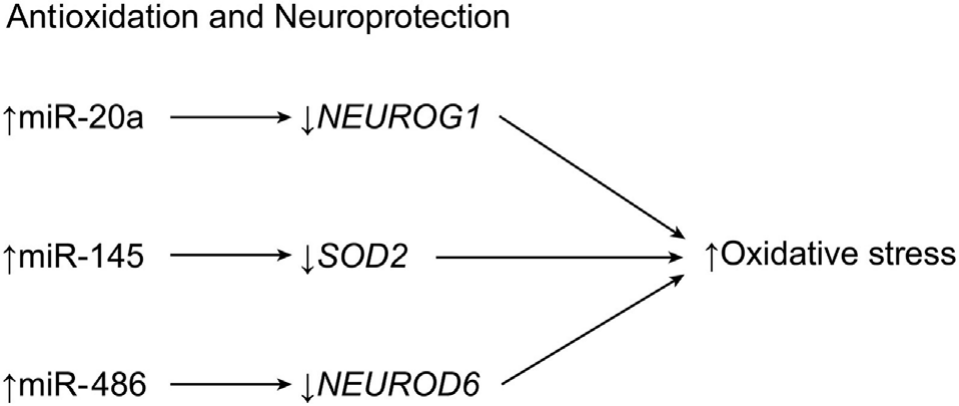
**Antioxidation and neuroprotection of central nervous tissue**. After SCI, increased expression of three miRNAs results in a loss of neuroprotective metabolites, causing reactive oxygen species-induced apoptosis. *NEUROD6*, neurogenic differentiation 6; *NEUROG1*, neurogenin 1; *SOD2*, superoxide dismutase 2. Used with permission from Barrow Neurological Institute, Phoenix, AZ, USA.

One example is miR-486, which targets the neuronal differentiation 6 gene, *NEUROD6*, a trigger for heat shock proteins. When miR-486 is knocked down, the expression of *NEUROD6* caused increased clearance of ROS and lower levels of proinflammatory agents (Figure 3) (7, 68). The neurogenin 1 gene, *NEUROG1*, is a differentiation factor in embryogenesis whose overexpression in progenitor cells heavily favors neuronal differentiation. It is generated from the same precursor factor that produces *NEUROD6* (Table 1) (7, 69, 70). The miRNA miR-20a, which is overexpressed in SCI, targets *NEUROG1* and prevents neuronal regrowth in the lesion site, presumably contributing to the motor neuron degeneration and apoptosis that follows spinal cord trauma (Figure 3) (22). A therapy that blocked miR-20a or that introduced exogenous *NEUROG1* would result in regeneration of neurons and improved functional deficit, making miR-20a a prime candidate for therapy (7, 68). Conversely, miR-29b is implicated in having antiapoptotic effects in ischemia by repressing the apoptotic *BH3* gene (Table 1) (22). A treatment strategy could take advantage of these effects by manipulating miR-20a and miR-29b in tandem to treat SCI (22).

Dharap et al. demonstrated that miR-145 targets *SOD2* after experiments with antagomir-145 led to higher levels of *SOD2* expression in mice with cortical ischemia (Table 1; Figure 3) (42). A similar pattern of expression has been observed in cortical ischemia and in plexus root avulsion, such that neurons ipsilateral to the lesion site express the highest levels of miRNA (71). This relationship suggests a unilateral miRNA response to SCI injuries. However, the contralateral side shows microglial activation in what is hypothesized to be a spillover of the changes observed on the opposite side (42, 71). Recent research has shown that miR-200c is another miRNA significantly altered by apoptotic events after SCI, as miR-200c upregulation was observed alongside the downregulation of its target, Fas-associated phosphatase-1 (*FAP1*), and, ultimately, the induction of *FAS* signaling (50).

### Inflammation

Although inflammation is part of the natural healing process, much of medicine is geared toward minimizing it or eliminating it, altogether. Inflammation is constantly regulated by the body’s innate immune system (43). In the setting of SCI, the role of inflammation is that of deleterious effects causing tissue compression and excessive cell death. One of the main functions of astrogliosis is to minimize inflammatory reactions in and around the lesion site to minimize the spread of secondary damage beyond the initial point of trauma. In SCI, the expressions of the various miRNAs that help regulate the body’s inflammatory processes are altered and are thus an important target for potential therapies.

**Figure 4:**
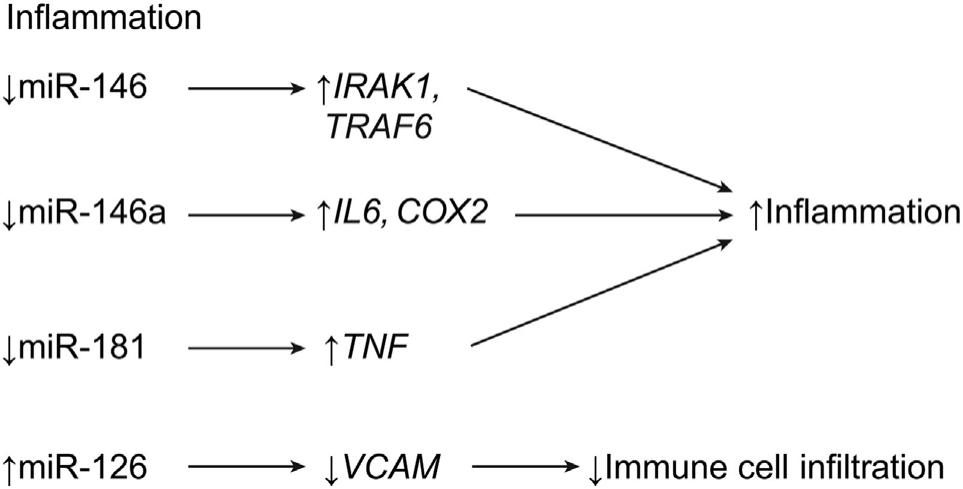
**Inflammatory effects of miRNA expression post SCI**. While downregulation of miR-146 and miR-181 leads to increased inflammation, upregulation of miR-126 leads to decreased extravasation of immune cells into the CNS. *COX2*, cyclooxygenase 2; *IL6*, interleukin 6; *IRAK1*, IL-1R-associated kinase 1; *TRAF6*, TNFR-associated factor 6; *TNF*, tumor necrosis factor; *VCAM1*, vascular cell adhesion molecule 1. Used with permission from Barrow Neurological Institute, Phoenix, AZ, USA.

The miRNA miR-146a is described above as being intimately cooperative with miR-21 and involved in the shift from astroglial hypertrophy to hyperplasia. Studies have shown that miR-146a is highly expressed in spinal astrocytes during SCI. It targets the proinflammatory enzyme cyclooxygenase-2 (COX-2) and the proteins encoded by the genes, *IL1B* and *IL6* (Table 1; Figure 4) (7, 18, 43, 44). In previous studies on temporal lobe epilepsy, astrocytes were highly expressed during latent periods, in relation to high levels of miR-146a (43, 44). Furthermore, these studies showed that miR-146a (in macrophages) works in cyclic feedback with the transcription factor NF-κB, *via* interleukin 1 receptor-associated kinase 1 gene (*IRAK1*) and the TNF receptor-associated factor 6 gene (*TRAF6*). Activation of the NF-κB pathway in macrophages upregulates miR-146a, resulting in the downregulation of *IRAK1* and *TRAF6* pathway constituents (Table 1) (43, 44). A correlation can be drawn between these phenomena and SCI, in which miR-146a levels peak and astrocytes increase anti-inflammatory activity. However, although some research on hyperplastic glial scars attributes functional deficit to miR-146a overexpression, other research deems miR-146a valuable in preventing the deleterious effects of inflammation (43, 44). Therefore, while miR-146a is beneficial in the schema of acute anti-inflammatory treatment, overexpression of miR-146a beyond the subacute stages of SCI seems to become deleterious, as seen with miR-21. The Notch-1 pathway may exhibit a potential therapeutic role in SCI. Notch-1 can lead to malignant astrocyte proliferation, but it is inhibited by miR-146 (44). This relationship is important, considering that previous research has implicated miR-146a in the negative effects of glial scar formation, which supports the idea that miR-146a attenuates the hyperplastic effects of miR-21 (44). Manipulation of miR-146a after SCI may allow for an ideal balance of anti-inflammatory and antihyperplastic effects, which may lead to improved healing and reduced scarring.

The miRNA miR-181 is expressed in macrophages, monocytes, and astrocytes (47, 48). It is a well-known anti-inflammatory agent because its overexpression leads to the suppression of proinflammatory cytokines, such as IL-1β, IL-6, IL-8, and TNFα. Furthermore, miR-181 suppresses HMGA-1, a proinflammatory factor that is regulated by *COX2* and *STAT3* (Table 1; Figure 4) (47, 48). However, miR-181 expression is decreased in SCI, which would allow for an increased inflammatory response after SCI. This situation is confounding in light of the strong anti-inflammatory signals that astrocytes send out during acute astrogliosis, which may mean that miR-181 is less functional in SCI than it is in Alzheimer’s disease or cortical ischemia, as research suggests (47). Another miRNA, miR-223, is shown to be overexpressed near areas of increased neutrophil aggregation, which suggests that miR-223 is implicated in neutrophil homing. This process counters that of hypertrophic astrocytes, which attempt to rid the central lesion of inflammatory cells during the subacute phase of astrogliosis (7, 54). The expression of miR-223 is time-dependent. It peaks twice at 12 h and 3 days after SCI and significantly decreases after that. This coincides with peak neutrophil expression 1 day after SCI, followed by downregulation 5 days after SCI (Table 1) (7).

In 2015, Hu et al. demonstrated that miR-126 is highly involved in attenuating inflammation, alongside angiogenesis and functional recovery (41). An agonist method showed that, in mice treated with exogenous miR-126, the expression of biomarkers for extravasated leukocytes and macrophages (CD45 and CD68, respectively) were downregulated. This observation implicates miR-126 as a potential therapeutic strategy for SCI, because it is downregulated in SCI. The miR-126 targets *VCAM1*, which is a receptor on endothelial cells for leukocyte homing. *VCAM1* was shown to be downregulated in agomir-126-treated mice (Table 1; Figure 4) (41).

## Processes of SCI Recovery Regulated by miRNA

### Neuroplasticity

One of the main clinical concerns after SCI is the potential for functional recovery. Chances for recovery after complete transverse spinal cord lesions are slim. When spinal cord lesions are incomplete, the chances of recovery are greater, in part due to the neuroplasticity of the cortical and subcortical neurons and glial cells (72). Chronic SCI can cause regional changes in glucose metabolism, revealed on positron-emission tomography as larger areas of activation in somatosensory regions for SCI patients compared with normal patients (72). Certain miRNAs are expressed in the brain, which indicates that miRNAs could function in both tissue development and higher brain function. In fact, researchers have compiled data that explain the array of morphological functions that miRNAs perform in the CNS (73, 74). An example of the functions of miRNAs in neuroplasticity is miR-133b in stroke. Xin et al. conducted an experiment in which mice with induced middle cerebral artery occlusion were infused with three types of modified murine mesenchymal stem cells: naïve, miR-133b(+), and miR-133b(−) (74). Their results showed that the greatest functional recovery occurred in subjects with the miR-133b(+), while no recovery occurred in miR-133b(−) subjects. Furthermore, it was determined that the miR-133b(+) mesenchymal stem cell group also exhibited the greatest increase in neuronal plasticity and neurite remodeling in the ischemic zone. Exosome-mediated transfer of mesenchymal stem cells occurred in greatest numbers to neurons and astrocytes, where miR-133 downregulated connective tissue growth factor expression (74). Connective tissue growth factor is colocalized with GFAP, which is highly expressed during hypertrophic astrogliosis. Selective expression of GFAP and not connective tissue growth factor during astrogliosis could potentially function to maximize the beneficial effects of hypertrophic astrocytes.

### Axon Regeneration and Remyelination

In mammals, regeneration is the main difference in recovery between a peripheral nervous system injury and a CNS injury. Peripheral nervous system injuries are more likely to self-repair, whereas CNS injuries do not self-repair. Peripheral nervous system axons have one Schwann cell per myelin sheath (equaling many Schwann cells per single axon), but CNS axons have one oligodendrocyte per several sheaths. Thus, oligodendrocytes are far more indispensable than Schwann cells. Therefore, the CNS is capable of regeneration, so long as the oligodendrocytes remain intact, which is often not the case in SCI, when trauma, ROS, inflammation, and other factors destroy any neuron or glial cell in their path.

**Figure 5:**
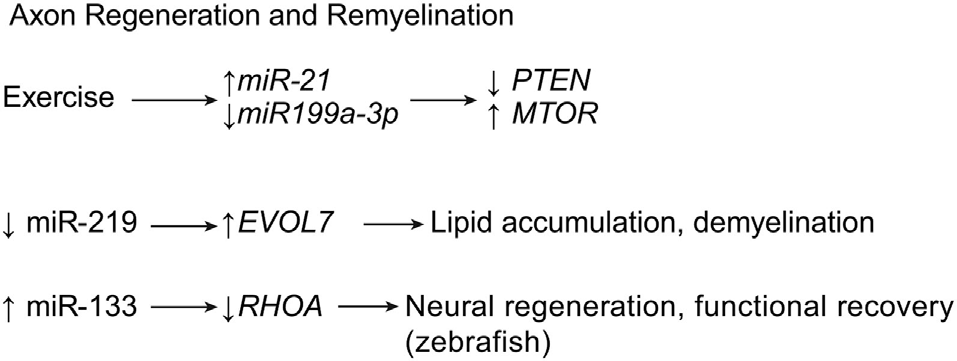
**Axon regeneration and remyelination by miRNA expression**. Exercise is known to decrease inflammation and thus increase regeneration of axon structures. Meanwhile, downregulation of miR-219 showed to induce demyelination after SCI. *ELOVL7*, elongation of very long chain fatty acids protein 7; *MTOR*, mechanistic target of rapamycin; *PTEN*, phosphatase and tensin homolog; *RHOA*, ras homolog gene family, member A. Used with permission from Barrow Neurological Institute, Phoenix, AZ, USA.

Recent research shows that it may be possible to rebuild oligodendrocytes and to repair axonal damage after SCI using miRNAs. Park et al. regenerated axons after optic nerve injury by deleting the phosphatase and tensin homolog gene, *PTEN*, and thereby upregulating the mechanistic target of rapamycin gene, *MTOR*, in the adult retinal ganglion cells of adult mice (26). As a follow-up to this article, Liu et al. extrapolated on their earlier work, in which early exercise after SCI correlated with upregulation of miR-21 and downregulation of miR199a-3p (Table 1) (27). According to Liu et al., the upregulation of miR-21 and the downregulation of miR199a-3p with exercise lead to the subsequent downregulation of *PTEN* and the upregulation of *MTOR*, the respective targets of miR-21 and miR-199a-3p (Figure 5) (27). This evidence strengthens the idea that bimodal control of neuronal apoptosis and axon degeneration can be achieved through these two miRNAs (Table 1) (26, 27).

Letzen et al. have shown that there are many miRNAs involved in the growth and proliferation of oligodendrocytes (28). Multiple oligodendrocyte-related miRNAs can be regulated by an oligodendrocyte-specific dicer. Research shows that mutant mice lacking the oligodendrocyte-specific dicer suffer brain demyelination and axonal impairment (Table 1) (53). These traits were found in addition to severe astrogliosis, inadequate antioxidant systems, and increased inflammation in the brain. Of the three major miRNAs downregulated by oligodendrocyte-specific dicer inhibition, miR-219 is observed to have the greatest contribution to astrogliosis, oxidative stress, and inflammation. Downregulating miR-219 leads to the dysregulation of its target gene, elongation of very long chain fatty acids protein 7 gene, *ELOVL7*, causing lipid accumulation in the white matter of the brain (Figure 5) (53). If miR-219 can be identified as playing an important role in the spinal cord, a potential therapy may be found in upregulating its expression after SCI.

The expression patterns of miRNA-125b help explain the discrepancies between the regenerative powers of reptiles and mammals (40). Reptiles are capable of regenerating CNS tissue after excision, whereas mammals are not. In the axolotl salamander, expression of miR-125 is precisely controlled, so that excess expression causes erratic axonal growth with incomplete reconnection, and reduced expression causes inhibition of axonal regeneration. In addition, glial scars are not seen after SCI in the axolotl, as they are in rats. This study shows that mammals could potentially benefit from increased miR-125 expression after SCI (40).

As mentioned above, the miRNA miR-133 was found to have a beneficial effect on neuroplasticity. Yu et al. demonstrated that miR-133 exhibits axonal regenerative properties in zebrafish, where it targets the ras homolog family member A gene, *RHOA*, whose product is a GTPase that inhibits neural regeneration and functional recovery in mammals and fish (Figure 5) (75). Another miRNA, miR-210, has been implicated as a possible therapeutic strategy for SCI, because it has been correlated with axon growth, in addition to neovascularization, astrogliosis, and myelination. The miR-210 suppresses the protein tyrosine phosphatase, non-receptor type 1 gene, *PTPN1*, and the ephrin-A3 gene, *EFNA3*, which has been shown to provide these benefits, leading to functional recovery in mice (Table 1) (51). As mentioned above, the miRNA miR-9 controls apoptotic factors during acute and subacute SCI (see Apoptosis). In addition to these effects, miR-9 has also been shown to play a role in suppressing Schwann cell migration, a critical step in neuroregeneration. Because of this ability, Xu et al. suggest that lower levels of miR-9 are needed during the acute stage of SCI to allow for adequate axon regeneration and remyelination (Table 1) (20).

### Neuron Regeneration

Even though the CNS is restorable after injury if oligodendrocytes are intact, neuron degeneration is a problem after SCI, because, so far, neurons cannot be brought back. However, miRNA therapy offers an avenue to achieve neuron regeneration.

**Figure 6:**
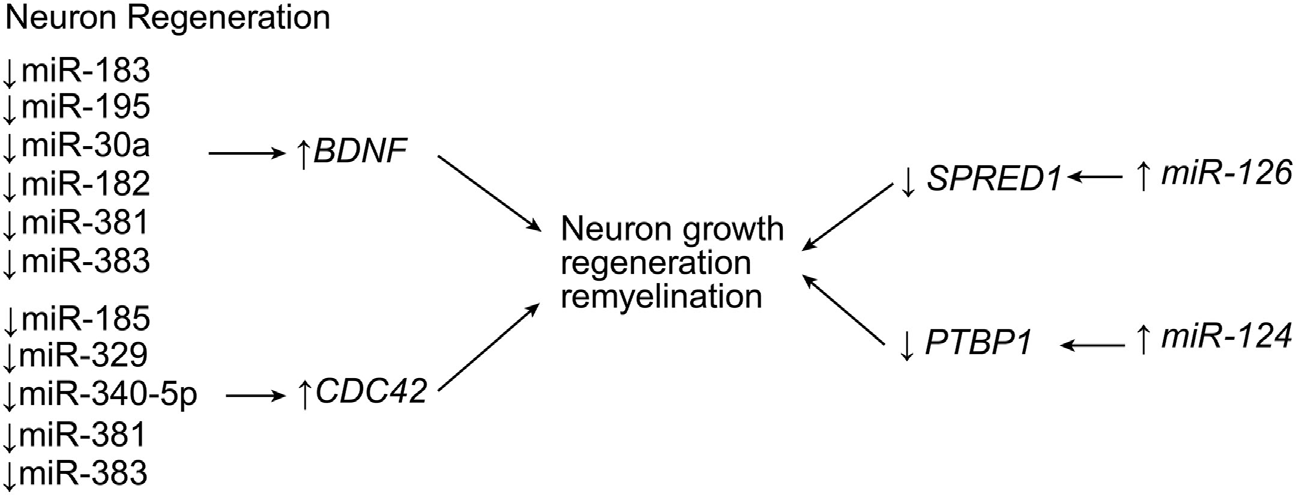
**Neuroregenerative effects of miRNA expression**. Multiple miRNA are responsible for neuron growth, regeneration, and remyelination. Upregulation of miR-126 and miR-124 leads to downregulation of negative regulators of growth signaling, while the downregulation of many other miRNA induce the expression of pro-growth factors. *BDNF*, brain-derived neurotrophic factor; *CDC42*, cell division protein 42; *PTBP1*, polypyrimidine tract-binding protein 1; *SPRED1*, Sprouty-related *EVH1* (enabled/vasodilator-stimulated phosphoprotein homology 1) domain containing 1. Used with permission from Barrow Neurological Institute, Phoenix, AZ, USA.

A bioinformatics study by Liu et al. (76) demonstrated that the body attempts to preserve neurons and to stimulate neuron growth, regeneration, and remyelination through the expression of a handful of genes through the action of miRNAs. Genes such as the brain-derived neurotrophic factor gene, *BDNF*, and the cell division cycle 42 gene, *CDC42*, are genes positively influencing SCI self-repair. The expression of these genes is inversely related to a large list of miRNAs that are thought to target them (*BDNF* can be influenced by miR-183, miR-195, miR-30a, miR-182, miR-381, miR-300-3p, and miR-325-5p; and CDC42 can be influenced by miR-185, miR-329, miR-340-5p, miR-381, and miR-383) (Table 1; Figure 6). Additionally, several miRNAs are thought to promote these two genes, and a balance between these two sets of miRNAs may play a critical role in self-repair (76). miR-124 restores neurons and recovers function (35). In SCI, miR-124 is downregulated continuously through the first 7 days after SCI at and around the lesion site (Table 1; Figure 6) (36, 37). Zou et al. observed that mesenchymal stem cells derived from bone marrow do not naturally express adequate levels of miR-124, so they transfected mesenchymal stem cells with miR-124, transplanted those cells into injured rat spinal cords, and observed that the transfected mesenchymal stem cells had significant neuronal expression (35). The transplanted rats had higher scores on the Basso, Beattie, and Bresnahan locomotor scale, and their functional recovery was higher, but their apoptosis was lower. These data suggest that the overexpression of miR-124 is linked with neural cell development and regeneration in SCI (35). miR-124 acts by targeting the polypyrimidine tract-binding protein 1 gene, *PTBP1*, which is a regulatory gene for neural precursor cell differentiation (36, 37). Xu et al. conducted a similar experiment; (38) however, they used neural stem cells from bone marrow-derived mesenchymal stem cells. After conducting a very similar experiment to that of Zou et al. (35), Xu et al. also observed significantly greater motor function outcomes in rats with SCI (38). It might be of interest for future studies to evaluate the time-dependence of therapy with miR-124 in order to understand its effects on astrogliosis since miR-124 selectively proliferates neurons over glial cells. Other studies have implicated miR-124 in reducing CNS inflammation by downregulating macrophage/microglia expression, while maintaining astrocytic expression, thus maintaining the anti-inflammatory effects of astroglial scarring (39).

Hu et al. made similar observations with miR-126, when they demonstrated that the injection of miR-126 increased protection of spinal cord motor axons (41). Typically, miR-126 is significantly downregulated by SCI, but upregulation therapy reversed this expression pattern, causing a decrease in apoptosis of motor neurons and an increase in functional motor recovery as long as 28 days after SCI (41). One of the main targets of miR-126, the phosphoinositide-3-kinase regulatory subunit 2 gene (*PIK3R2*) is a negative regulator of the phosphoinositide 3-kinase (PI3K) pathway, and thus, the apoptotic pathway. Another target is the sprouty-related, EVH1 domain-containing 1 gene (*SPRED1*), which is a negative regulator of growth factor signaling (Table 1; Figure 6) (41).

Neural stem cells have been a therapeutic strategy focus. Shi et al. demonstrated that miR-381 regulates neural stem cell differentiation (56). They found that overexpression of miR-381 in neural stem cells causes the expression of the hes family bHLH transcription factor 1 gene, *HES1*, which triggers proliferation and differentiation into neurons, but which inhibits differentiation into astrocytes (Table 1). These results suggest that manipulating neural cell differentiation should be done in a time-dependent manner, depending on the length of time since the patient’s injury. Introducing miR-381-infused neural stem cells and inhibiting astrocyte proliferation during acute stages of SCI may inhibit the beneficial effects of astrogliosis during acute stages. Conversely, infusing miR-381 during subacute or chronic stages may be of great benefit in reversing the cell death seen later.

### Functional Recovery

**Figure 7:**
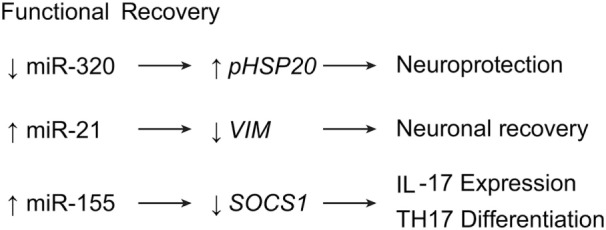
**miRNA expression leading to functional recovery after SCI**. Downregulation of miR-320 leads to neuroprotective effects, while upregulation of miR-21 and miR-155 induce neuronal recovery and T-cell expression, respectively. *IL17*, interleukin 17; *HSP20*, heat shock protein 20; *SOCS1*, supressor of cytokine signaling 1; Th17, T helper cell 17. Used with permission from Barrow Neurological Institute, Phoenix, AZ, USA.

The main objective in researching SCI is ultimately to help patients achieve superior functional recovery. Importantly, investigators often achieve functional recovery in experiments on miRNAs in SCIs (7, 15, 16, 25, 29, 30, 35, 38, 47, 51, 72, 74, 75, 77, 78). Recent evidence shows miRNAs may be helpful for repairing hind limb functionality in murine SCI (55). In this study, inhibitory treatment of miR-320 markedly improved motor scores, while upregulating the expression of phosphorylated *HSP20*, a gene that protects against ischemia-reperfusion injury (Table 1; Figure 7) (55).

A view not previously mentioned is observed with vimentin in scar formation. GFAP and vimentin are factors expressed in hypertrophying astrocytes, and their expressions are downregulated by miR-21 during the shift toward hyperplastic astrogliosis (14–16). In contrast, Qian et al. demonstrated highly expressed vimentin levels in spinal cord scar tissue as a result of astrocytic hyperplasia. In fact, the knockdown of vimentin in scar tissue showed significant improvement in locomotor function, while overexpression of vimentin showed the opposite (Table 1; Figure 7) (77). This result suggests that vimentin might play a detrimental role in astrogliosis, as opposed to our previous understanding.

Research on miR-155 has provided insight into another possibility for functional recovery from SCI. The miR-155 is normally upregulated in most leukocytes; it is also upregulated during SCI, contributing to the inflammatory destruction of the spinal cord. However, in miR-155 knockout mice, Basso Mouse Scale locomotor scores have been found to be higher 6 weeks after SCI compared with control groups. In addition, miR-155 knockout mice also expressed significantly lower levels of IL-17–expressing T cells, suggesting that miR-155 has a direct effect on Th17 cell proliferation after SCI *via* the regulation of Janus-associated kinase/signal transducer and activator of transcription (JAK/STAT) signaling by the suppressor of cytokine signaling 1 gene, *SOCS1* (Table 1; Figure 7) (45, 46).

Spinal cord injury is often associated with severe pain syndrome. Therefore, careful consideration should be given to optimal pain treatment of patients with SCI, as 1 mode in particular leads to decreased motor recovery and increased chronic pain (30). In their study of morphine delivery and SCI, Strickland et al. noticed that acute administration of morphine correlated to these effects, potentially *via* the expression of miRNA, including miR-21 and 146 (30). The miR-21 and 146 expressions were elevated by morphine. However, the rats experienced significant dysregulation of miR-21 and decreased motor function up to 15 days after SCI recovery. These results may be attributable to morphine-induced inflammation. They further suggest that the effects of miR-21 on SCI recovery are time-sensitive and must be regulated beyond the acute stages of SCI, both to optimize the beneficial effects of miR-21 and to minimize its deleterious effects.

### Pain

Increasing evidence over the past decade has suggested that miRNAs play a significant role in regulating both inflammatory and neuropathic pain following SCI (18, 29, 32–34, 49, 79–87). One of the major objectives in identifying miRNA sequences that influence pain modulation is to manipulate them for therapeutic uses. Although many miRNA sequences have already been found to influence chronic pain at the site of the lesion and in higher cortical structures, only a few miRNA sequences have been modulated and have been confirmed to have a therapeutic effect (33, 34, 49, 83, 86, 87).

**Figure 8:**
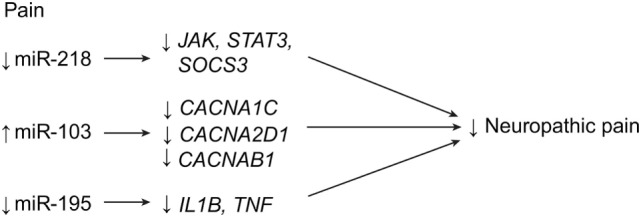
**Pain regulation with miRNA expression after SCI**. Downregulation of miR-218 and miR-195, in addition to upregulation of miR-103, lead to decreases in neuropathic pain after SCI. *CACNA1C*, calcium channel, voltage gated, L-type, alpha-1 C; *CACNA2D1*, calcium channel voltage gated, L-type, alpha-2 delta-1; *CACNB1*, calcium channel, voltage gated, L-type, beta-1; *IL1B*, interleukin 1 beta; *JAK*, Janus kinase; *STAT3*, signal transducer and activators of transcription 3; *SOCS3*, supressor of cytokine signaling 3; *TNF*, tumor necrosis factor. Used with permission from Barrow Neurological Institute, Phoenix, AZ, USA.

In 2016, Li and Zhao demonstrated that miR-218 expression is consistent with neuropathic pain symptoms in rats with compressive spinal cord injuries (52). When miR-218 was downregulated in rats, pain behavior and inflammation decreased. The authors hypothesize that miR-218 acts by inhibiting the JAK/STAT3 pathway by influencing the expression of the suppressor of the cytokine signaling 3 gene, *SOCS3* (Table 1; Figure 8) (52). In 2011, Favereaux et al. identified miR-103 as regulating three subunits of a L-type voltage-gated calcium channel named CaV1.2 (subunits alpha-1C, alpha-2 delta-1, and beta-1 are encoded by *CACNA1C, CACNA2D1*, and *CACNB1*, respectively) in dorsal horn neurons. This regulation was found to be bidirectional: upregulation of miR-103 led to downregulation of each subunit, and vice versa (Table 1; Figure 8) (33). This finding was significant because an earlier study had already established a strong connection between the CaV1.2 protein and chronic pain, showing that a knockout of CaV1.2 protein would lead to complete reversal of long-term sensitization (32, 34). Favereaux et al. also showed that the intrathecal application of miR-103 significantly relieved pain, thus establishing miR-103 as a strong candidate for the treatment of chronic pain after SCI (33).

Another miRNA that has been linked to inflammatory pain after SCI is miR-195 (49). It was found to increase significantly after spinal nerve ligation, and it was also associated with the continuous release of proinflammatory cytokines, such as IL-1β and TNF-α (Figure 8). These researchers proposed that upregulation of miR-195 leads to decreased autophagic activity and thus to increased neuroinflammation. In addition, upregulation of miR-195 was also positively linked with increased mechanical and cold sensitivity (49).

Both miR-103 and miR-195 have been shown to modulate chronic pain at the lesion site (33, 34, 49). Substantial evidence also shows that changes in miRNA expression in higher cortical structures are associated with inflammatory and neuropathic pain, indicating that pain relief therapies for SCI may not be restricted to the site of injury and nearby structures (83–87). For instance, miRNA expression changes substantially in both the prefrontal cortex and the hippocampus when stimulated by chronic pain (83, 86, 87).

Although the role of miRNAs in pain regulation is substantial, the exact mechanisms involved in pain sensation are very complex and require much further study (32). However, preliminary data suggest a promising role for the use of miRNAs as therapeutic agents for pain relief in many pathologic conditions, including SCI (32).

## Conclusion

Spinal cord injury is a serious and debilitating injury with limited treatment resources. After the initial injury to the spinal cord, numerous secondary pathophysiological events occur that contribute to a major part of the total damage. Such secondary events include inflammation, apoptosis, ROS formation, and astrogliosis. In recent years, many studies have identified miRNAs as contributors and regulators of secondary injury, with most of the research providing specific mRNA targets for the miRNA involved. Not all miRNAs affect SCIs negatively, however. Some miRNAs appear to promote the beneficial aspects of the healing mechanisms of the body. For instance, miRNAs have been shown to promote neuroplasticity, axon regeneration and remyelination, neuron cell regeneration, and functional recovery. However, the underlying problem is that, in most cases, SCI causes both the overexpression of harmful miRNAs and the inhibition of beneficial miRNAs. Manipulating the expression of miRNAs after SCI might be a new therapeutic strategy for overcoming the lasting and detrimental effects of SCI, thereby giving clinicians better diagnostic tools and giving patients better outcomes. Overall, miRNAs may lead to an era of personalized medicine for individuals with SCIs. More research is mandated, and the expected results should provide new hope for better treatment of patients with SCIs.

## Author Contributions

All authors made substantial contributions to the conception or design of the work.

## Conflict of Interest Statement

The authors declare that the research was conducted in the absence of any commercial or financial relationships that could be construed as a potential conflict of interest.

## Abbreviations

BBB, blood–brain barrier; BMP, bone morphogenic protein; CNS, central nervous system; GFAP, glial fibrillary acidic protein; JAK/STAT, Janus-associated kinase/signal transducer and activator of transcription; miRNA, microRNA; ROS, reactive oxygen species; SCI, spinal cord injury; SOD, superoxide dismutase.
